# Sunglasses use, UV protection, and knowledge of UV radiation among adults in Poland: a cross-sectional study

**DOI:** 10.13075/ijomeh.1896.02907

**Published:** 2026

**Authors:** Agnieszka Kamińska, Radosław Sierpiński, Agata Olearczyk, Aysel Galbinur, Tural Galbinur, Maciej Kamiński, Mariusz Gujski, Mateusz Jankowski, Grzegorz Juszczyk

**Affiliations:** 1 Cardinal Stefan Wyszynski University, Faculty of Medicine, Collegium Medicum, Warsaw, Poland; 2 Cardinal Stefan Wyszynski University, National Centre for Health Policy and Health Inequalities, Warsaw, Poland; 3 Azerbaijan Medical University, Department of Ophthalmology, Baku, Azerbaijan; 4 Łazarski University, Faculty of Medicine, Warsaw, Poland; 5 VIZJA University, Warsaw, Poland; 6 Centre of Postgraduate Medical Education, School of Public Health, Warsaw, Poland; 7 Medical University of Warsaw, Department of Public Health, Faculty of Health Sciences, Warsaw, Poland

**Keywords:** health awareness, population-based study, ultraviolet radiation, UV protection, eye health, sunglasses use

## Abstract

**Objectives:**

Ultraviolet (UV) radiation (UVR) is an environmental risk factor for several ocular conditions. This study aimed to assess sunglasses use patterns and awareness of UV-related eye health risks, as well as to identify sociodemographic factors associated with sunglasses use among adults in Poland.

**Material and Methods:**

A nationwide cross-sectional survey was conducted in March 13–17, 2026 among 1090 adults in Poland. Data were collected using the computer-assisted web interview technique. A quota-based sample was used. Sunglasses use, UV protection, outdoor exposure, knowledge of UVR, and barriers to use were assessed.

**Results:**

Frequent sunglasses use was reported by 58.5% of respondents on sunny spring/summer days, but only by 23.5% on cloudy spring/summer days. Less than half of respondents (44.2%) declared using sunglasses with UV protection, while 22.3% did not know whether their sunglasses provided such protection. Overall knowledge of UVR was relatively good, although 47.2% incorrectly believed that darker lenses provide better UV protection. In multivariable logistic regression, female gender (adjusted odds ratio [aOR] = 1.82, 95% confidence interval [CI]: 1.41–2.34, p < 0.001), occupational activity (aOR = 1.84, 95% CI: 1.35–2.52, p < 0.001), living in cities <20 000 residents (aOR = 1.57, 95% CI: 1.04–2.35, p = 0.03), and having a doctor-diagnosed eye condition (aOR = 1.45, 95% CI: 1.11–1.90, p = 0.007) were significantly associated with frequent sunglasses use on sunny days during spring and summer. Older age (40–49 years: aOR = 1.71, 95% CI: 1.08–2.72, p = 0.02; 50–59 years: aOR = 1.93, 95% CI: 1.22–3.05, p = 0.005), higher education (aOR = 1.49, 95% CI: 1.15–1.94, p = 0.002), occupational activity (aOR = 1.79, 95% CI: 1.28–2.51, p < 0.001), living in the largest cities (≥500 000 residents: aOR = 1.79, 95% CI: 1.20–2.68, p = 0.005), and having a doctor-diagnosed eye condition (aOR = 1.67, 95% CI: 1.28–2.17, p < 0.001) were significantly associated with the use of sunglasses with UV protection.

**Conclusions:**

Sunglasses use among adults in Poland is insufficient, particularly under cloudy conditions, and misconceptions regarding UV protection remain common. These findings indicate the need for targeted public health interventions to improve awareness and promote appropriate UV eye protection.

## Highlights

- Sunglasses use was low during cloudy spring and summer days.
- Only 44.2% respondents used sunglasses with ultraviolet (UV) protection.
- Nearly half respondents believed darker lenses provide better UV protection.
- Older age, higher education, and eye disease were associated with UV protection use.
- Targeted UV eye protection education campaigns are urgently needed.

## INTRODUCTION

Ultraviolet (UV) radiation (UVR) is a well-established environmental risk factor linked to several adverse outcomes, including skin aging, immunosuppression, and increased risk of skin cancers, especially melanoma [1–5]. Except well-documented effects on skin, UVR also poses a significant risk to eye health [3–5]. Chronic exposure to UVR has been associated with ocular conditions like cataract, pterygium and age-related macular degeneration (AMD) [3–5]. Moreover, UVR may cause acute eye conditions such as photokeratitis [3–6].

Ultraviolet radiation is usually divided into 3 ranges: UV-A (315–400 nm), UV-B (280–315 nm), and UV-C (100–280 nm). The primary components relevant to eye health are UV-A and UV-B, as UV-C is almost completely absorbed in the atmosphere and does not reach the Earth's surface [7,8]. Ultraviolet A penetrates into the eye and may induce oxidative stress that causes long-term degenerative process. Ultraviolet B radiation is absorbed by the cornea and lens poses short-term (photekeratitis) and long-term (e.g., cataract formation) threats to eye health [5–8]. Ultraviolet radiation poses a threat for eye health not only in sunny days, but also under such weather conditions as reduced sunlight, cloudy days or reflection from surfaces like snow or water. These factors contribute to cumulative ocular UV exposure throughout the year, not only during periods of intense sunlight [5,6,8].

Wearing sunglasses with appropriate UV-blocking filters is considered the most effective measure for protecting the eyes against UVR. Filters such as UV400 block nearly 100% of UV-A and UV-B radiation. Protection against UVR does not depend on lens tint or darkness of the lenses [9–11]. Correct and consistent use of sunglasses with adequate UV protection is therefore essential to effectively reduce cumulative ocular exposure to UVR and reduce risk of UVR-related eye conditions [9–12].

Scientific data suggest that preventive behaviors aimed at reducing UV exposure to the eyes remains insufficient. The regular use of sunglass varies 30–60% of adults using sunglasses during sunny conditions. In cloudy conditions the prevalence of sunglasses use is significantly lower. Awareness of UV-related health conditions is often low, with substantial knowledge gaps related to exposure and potential health consequences [13–18]. Moreover, misconceptions, e.g., on the darker lenses providing better protection and limited exposure to UVR during cloudy days are quite common [16–18]. Despite the significant impact of UVR on eye health, there are limited population-based data on public attitudes towards preventive measures aimed at UVR protection with sunglasses equipped with UV filters [13–18].

Poland, is a Central European country with a temperate climate, UV exposure is characterized by pronounced seasonal variability, with low levels during winter and substantially higher levels from late spring to early autumn [19]. Ultraviolet exposure also occurs during winter as a consequence of UV reflection from the snow [20]. These conditions contribute to cumulative exposures that may be underestimated by the population [19,20].

Despite the significant influence of environmental factors on human health, media campaigns on eye protection from UVR remain limited [21]. There is a lack of population-based data on protective behaviors and knowledge on UVR and eye health in Poland [15]. Moreover, evidence on sociodemographic determinants of these behaviors is scarce, highlighting the need for studies that can inform targeted public health interventions. Therefore, this study aimed to assess sunglasses use patterns and awareness of UV-related eye health risks, as well as to identify sociodemographic factors associated with sunglasses use among adults in Poland.

## MATERIAL AND METHODS

### Study design and population

This cross-sectional survey was carried out 13–17 March 2026 among a nationwide sample of adults in Poland. Data were collected using computer assisted web-interview (CAWI) technique. Data collection process was conducted by public opinion survey company (Nationwide Research Panel Ariadna, Warsaw, Poland), based on a study protocol and methods provided by the authors [22].

Participants were selected from an online research panel of >100 000 registered and verified individuals [22]. A quota-based sampling was applied, with stratification model including gender, age and place of residence. The sampling structure reflected the demographic distribution of the Polish adult population, based on the official demographic data published by Statistics Poland [23]. In Poland, 96% of households has an Internet access, so the CAWI technique used in this study allowed for obtaining nationwide sample of adults in Poland.

Invitations to participate in this study were distributed via email and SMS. Each respondents received an individualized link to the online questionnaire hosted on a secured platform managed by the Nationwide Research Panel Ariadna [22]. In case of non-response, participants matching sociodemographic characteristics were invited to maintain the predefined quotas. All questionnaires were fully completed so there were no missing answers.

Methods applied in this study are consisted with procedures used in previous nationwide survey studies conducted in Poland [24–26].

According to the survey company, the estimated average response rate of the Nationwide Research Panel Ariadna is approx. 22%.

### Questionnaire

The study questionnaire consisted of 16 questions addressing eye health and preventive behaviors. It was prepared based on a literature review [13–21] and included items assessing outdoor exposure, sunglasses use patterns, UV protection, and knowledge of UVR and eye health.

Daily time spent outdoors during spring and summer months (approx. from early April to the end of September) was measured using a single-choice question with 6 response categories ranging from “<30 min/day” to “>6 h/day”.

Frequency of sunglasses use in different weather and seasonal conditions was assessed using a 5-point Likert scale (never, rarely, often, very often, always), separately for sunny and cloudy days in spring/summer and autumn/winter. For the purpose of statistical analysis, respondents who selected “often,” “very often,” or “always” were classified as individuals regularly wearing sunglasses. Participants were also asked whether the sunglasses they usually use provide UV protection (yes, no, do not know, do not own sunglasses).

Among respondents declaring the use of UV-protective sunglasses, the source of this knowledge was assessed using a multiple-choice question. Participants who did not use UV-protective sunglasses or were unsure were asked about barriers to their use, using a multiple-response question including lack of need, cost, discomfort, and uncertainty about product quality.

Knowledge of UVR and eye protection was evaluated using a series of statements rated on a 5-point Likert scale (strongly disagree, rather disagree, rather agree, strongly agree, and difficult to say).

A pilot study was conducted in a group of 18 adults, who completed the questionnaire twice with a 7-day interval. Based on this pre-test, 4 items were revised to improve clarity and consistency. The κ coefficients for key items ranged 0.64–0.92.

### Ethics

This study followed the rules expressed in Declaration of Helsinki. The study protocol was verified and approved by the Ethics Committee at the Center of Postgraduate Medical Education (decision No. 3/2026 as of February 11, 2026). Participation in this study was voluntary and anonymous. Informed consent were collected from all the participants.

### Statistical analysis

Analyses were conducted using IBM SPSS Statistics v. 29 (IBM, Armonk, NY, USA). Categorical variables were presented with frequencies and proportions. Cross-tabulations and χ^2^ tests were used for the comparison of categorical variables. Logistic regression analyses were performed to identify factors associated with frequent sunglasses use and the use of sunglasses with UV protection. In the first step, bivariable logistic regression models were applied for each independent variable. Statistically significant variables were subsequently included in multivariable logistic regression models. Results were reported as odds ratios (ORs) with 95% confidence intervals (CIs). A p-value of <0.05 was considered statistically significant.

## RESULTS

Data were collected from 1090 adults aged ≥18 years, 54.1% were females (Table 1). Most respondents lived in rural areas (39.6%), were occupationally active (61.5%), and assessed their financial status as good or moderate (85.7%). A majority reported wearing spectacles or contact lenses (69.3%), while 35.4% had at least one eye condition diagnosed by a doctor (Table 1).

**Table 1.** Characteristics of the study population in a nationwide cross-sectional survey on sunglasses use, UV protection, outdoor exposure, knowledge of UVR, and barriers, Poland, 2026

Variable	Participants (N = 1090)	
	n	%
Socioeconomic		
gender		
female	590	54.1
male	500	45.9
age		
18–29 years	139	12.8
30–39 years	220	20.2
40–49 years	205	18.8
50–59 years	203	18.6
≥60 years	323	29.6
higher education		
yes	500	45.9
no	590	54.1
currently married		
yes	586	53.8
no	504	46.2
place of residence		
rural area	432	39.6
city		
<20 000 residents	142	13.0
20 000–99 999 residents	207	19.0
100 000–499 999 residents	171	15.7
≥500 000 residents	138	12.7
having children <18 years		
yes	275	25.2
no	815	74.8
number of household members		
1 (living alone)	178	16.3
2	397	36.4
≥3	515	47.2
occupational status		
active	670	61.5
passive	420	38.5
self-declared financial status		
good	510	46.8
moderate	424	38.9
bad	156	14.3
Medical		
wearing spectacles or contact lenses		
yes	755	69.3
no	335	30.7
eye condition diagnosed by a doctor		
yes	386	35.4
no	704	64.6

### Sunglasses use and knowledge about UVR

Respondents most often reported wearing sunglasses on sunny days in spring and summer – 58.5% declared using them often, very often, or always (compared to 38.3% declaring using them on sunny days in winter and autumn), while use was much lower on cloudy days (23.5% in spring/summer and 18.3% in autumn/winter) (Table 2). Overall knowledge about UVR was relatively good, with the majority agreeing that UV reaches the eyes on cloudy days (74.1%), that snow reflection increases exposure (75.5%), and that UV exposure is higher in mountainous areas (75.1%). However, some misconceptions persisted, as 47.2% believed that darker lenses provide better UV protection (Table 2).

**Table 2.** Sunglasses use patterns and knowledge about ultraviolet (UV) radiation (UVR) and eye protection, nationwide cross-sectional survey, Poland, 2026

Variable	Participants N = 1090 [n (%)]									
	sunglasses usage					acceptance of a statement				
	never	rarely	often	very often	always	strongly disagree	rather disagree	rather agree	strongly agree	difficult to say
Weather and seasonal conditions										
on a sunny day in spring and summer	219 (20.1)	234 (21.5)	272 (25.0)	228 (20.9)	137 (12.6)					
on a cloudy day in spring and summer	549 (50.4)	285 (26.1)	150 (13.8)	73 (6.7)	33 (3.0)					
on a sunny day in winter and autumn	389 (35.7)	283 (26.0)	227 (20.8)	120 (11.0)	71 (6.5)					
on a cloudy day in winter and autumn	671 (61.6)	219 (20.1)	118 (10.8)	57 (5.2)	25 (2.3)					
Knowledge about UVR and eye protection										
UVR can still reach the eyes on a cloudy day						17 (1.6)	47 (4.3)	514 (47.2)	293 (26.9)	219 (20.1)
reflection of sunlight from snow increases eye exposure to UVR						17 (1.6)	43 (3.9)	400 (36.7)	423 (38.8)	207 (19.0)
UVR exposure may be higher in mountainous areas than at lower altitudes						11 (1.0)	33 (3.0)	409 (37.5)	410 (37.6)	227 (20.8)
darker lenses always provide better protection against UVR						72 (6.6)	210 (19.3)	356 (32.7)	158 (14.5)	294 (27.0)
UV protection in sunglasses depends primarily on the filter (e.g., UV400), not on the level of lens tint						11 (1.0)	36 (3.3)	433 (39.7)	336 (30.8)	274 (25.1)
long-term exposure to UVR may contribute to the development of certain eye diseases						14 (1.3)	36 (3.3)	447 (41.0)	382 (35.0)	211 (19.4)
intense UV exposure (e.g., in mountains or on snow) may lead to acute damage to the eye surface (“snow blindness”)						14 (1.3)	29 (2.7)	418 (38.3)	415 (38.1)	214 (19.6)

### Outdoor exposure, UV protection and barriers to sunglasses use

Most respondents spent 1–4 h outdoors daily during spring and summer (58.3%), while only 6.2% reported <30 min (Table 3). Less than half (44.2%) declared using sunglasses with UV protection, and as many as 22.3% did not know whether their sunglasses provided such protection. Among those not using UV-protective sunglasses, the predominant reason was a lack of perceived need (81.4%), with fewer respondents indicating high cost (16.4%) or uncertainty about which products are reliable (10.7%) as barriers (Table 3).

**Table 3.** Outdoor exposure, ultraviolet (UV) protection in sunglasses, sources of knowledge, and barriers to use, nationwide cross-sectional survey, Poland, 2026

Question	Participants	
	n	%
On average, how much time per day do you spend outdoors during the spring and summer months (approximately from early April to the end of September)? (N = 1090)		
<30 min/day	68	6.2
30–59 min/day	152	13.9
1–2 h/day	320	29.4
3–4 h/day	315	28.9
5–6 h/day	189	17.3
>6 h/day	46	4.2
Do the sunglasses you usually use have UV protection? (N = 1090)		
yes	482	44.2
no	98	9.0
I do not know	243	22.3
I do not own sunglasses	57	5.2
I never use sunglasses	210	19.3
How do you know that your sunglasses provide UV protection? (N = 482)		
information on the frame or label (e.g., UV400)	188	39.0
assurance from the vendor (e.g., at a market stall)	47	9.8
purchased from an optical store with a UV protection guarantee	198	41.1
certificates or markings (e.g., CE)	163	33.8
difficult to say (not randomized)	17	3.5
Why do you not use sunglasses with UV protection? (all applicable reasons or others specified) (N = 544)		
I do not feel the need	443	81.4
sunglasses are uncomfortable (e.g., pressure on the nose, general discomfort)	45	8.3
the cost of UV-protective sunglasses is too high	89	16.4
I am concerned that UV filters may worsen vision or contrast	17	3.1
I see worse and less clearly when wearing UV-protective sunglasses	23	4.2
I do not know which brands or UV filters are reliable/safe	58	10.7
the selection is too wide and I find it difficult to choose	30	5.5
I find it difficult to fit sunglasses to my face	28	5.1
I use a hat or cap instead of sunglasses	37	6.8
I am rarely exposed to sunlight	35	6.4
other reasons	9	1.7
difficult to say	201	36.9

### Factors associated with sunglasses use in spring and summer months

Significant sociodemographic differences in sunglasses use were observed (Table 4). In multivariable logistic regression the following factors were significantly associated with higher odds of sunglasses use on sunny days during spring and summer:

– female gender (adjusted OR [aOR] = 1.82, 95% CI: 1.41–2.34, p < 0.001),
– living in cities <20 000 residents (aOR = 1.57, 95% CI: 1.04–2.35, p = 0.03) or in larger cities ≥100 000 residents (p < 0.05),
– occupational activity (aOR = 1.84, 95% CI: 1.35–2.52, p < 0.001),
– having a doctor-diagnosed eye condition (aOR = 1.45, 95% CI: 1.11–1.90, p = 0.007).

**Table 4.** Factors associated with frequent sunglasses use under sunny and cloudy conditions during spring and summer and with using sunglasses with ultraviolet (UV) protection among adults, nationwide cross-sectional survey, Poland, 2026

Variable	Respondents [n]	Wearing sunglasses in spring and summer – often, very often or always												Using sunglasses with UV protection					
		on a sunny day						on a cloudy day											
		participants (N = 1090)		bivariable logistic regression		multivariable logistic regression		participants (N = 1090)		bivariable logistic regression		multivariable logistic regression		participants (N = 1090)		bivariable logistic regression		multivariable logistic regression	
		%	p	OR (95% CI)	p	aOR (95% CI)	p	%	p	OR (95% CI)	p	aOR (95% CI)	p	%	p	OR (95% CI)	p	aOR (95% CI)	p
Socioeconomic																			
gender																			
female	590	64.7	**<0.001**	1.77 (1.38–2.25)	<0.001	1.82 (1.41–2.34)	**<0.001**	25.3	0.1	1.24 (0.94–1.65)	0.1			45.9	0.2	1.16 (0.92–1.48)	0.2		
male	500	51.0		ref.		ref.		21.4		ref.				42.2		ref.			
age																			
18–29 years	139	69.1	**0.001**	1.87 (1.22–2.84)	0.004	1.58 (0.99–2.50)	0.05	33.8	**<0.001**	2.66 (1.68–4.22)	<0.001	2.10 (1.25–3.53)	**0.005**	33.8	**0.01**	ref.		ref.	
30–39 years	220	59.5		1.23 (0.87–1.74)	0.2	0.89 (0.59–1.34)	0.6	31.4		2.38 (1.58–3.59)	<0.001	1.65 (0.99–2.74)	0.05	46.4		1.69 (1.09–2.63)	0.02	1.34 (0.84–2.12)	0.2
40–49 years	205	64.9		1.54 (1.08–2.21)	0.02	1.22 (0.79–1.87)	0.4	27.3		1.96 (1.28–3.00)	0.002	1.46 (0.84–2.52)	0.2	49.3		1.90 (1.22–2.97)	0.005	1.71 (1.08–2.72)	**0.02**
50–59 years	203	49.8		0.83 (0.58–1.18)	0.3	0.65 (0.44–0.97)	**0.04**	15.8		0.98 (0.60–1.58)	0.9	0.80 (0.47–1.38)	0.4	49.8		1.94 (1.24–3.03)	0.004	1.93 (1.22–3.05)	**0.005**
≥60 years	323	54.5		ref.		ref.		16.1		ref.		ref.		40.6		1.34 (0.88–2.02)	0.2	1.61 (1.01–2.57)	**0.04**
higher education																			
yes	500	60.8	0.1	1.20 (0.94–1.53)	0.1			23.8	0.8	1.03 (0.78–1.37)	0.8			50.8	**<0.001**	1.64 (1.29–2.09)	<0.001	1.49 (1.15–1.94)	**0.002**
no	590	56.4		ref.				23.2		ref.				38.6		ref.		ref.	
currently married																			
yes	586	57.7	0.6	0.93 (0.73–1.19)	0.6			22.9	0.6	0.93 (0.70–1.23)	0.6			46.8	0.07	1.25 (0.98–1.59)	0.07		
no	504	59.3		ref.				24.2		ref.				41.3		ref.			
place of residence																			
rural area	432	53.7	**0.002**	ref.		ref.		22.0	0.1	ref.				39.8	**0.002**	ref.		ref.	
city																			
<20 000 residents	142	64.1		1.54 (1.04–2.28)	0.03	1.57 (1.04–2.35)	**0.03**	28.9		1.44 (0.94–2.21)	0.1			43.0		1.14 (0.78–1.67)	0.5	1.14 (0.77–1.69)	0.5
20 000–99 999 residents	207	52.7		0.96 (0.69–1.34)	0.8	0.94 (0.66–1.33)	0.7	19.8		0.88 (0.58–1.32)	0.5			41.1		1.05 (0.75–1.48)	0.8	0.97 (0.69–1.38)	0.9
100 000–499 999 residents	171	64.9		1.60 (1.11–2.30)	0.01	1.58 (1.08–2.32)	**0.02**	22.8		1.05 (0.67–1.60)	0.8			49.1		1.46 (1.02–2.08)	0.04	1.32 (0.92–1.91)	0.1
≥500 000 residents	138	68.1		1.84 (1.23–2.76)	0.003	1.75 (1.15–2.67)	**0.01**	29.0		1.45 (0.94–2.23)	0.09			58.0		2.09 (1.41–3.08)	<0.001	1.79 (1.20–2.68)	**0.005**
having children <18 years																			
yes	275	60.7	0.4	1.14 (0.86–1.50)	0.4			29.1	**0.01**	1.49 (1.09–2.03)	0.01	1.17 (0.81–1.67)	0.4	49.1	0.06	1.30 (0.99–1.71)	0.06		
no	815	57.7		ref.				21.6		ref.		ref.		42.6		ref.			
number of household members																			
1 (living alone)	178	58.4	0.9	ref.				19.7	0.2	ref.				37.1	0.1	ref.			
2	397	58.9		1.02 (0.71–1.46)	0.9			22.2		1.16 (0.75–1.81)	0.5			45.8		1.44 (1.00–2.06)	0.05		
≥3	515	58.1		0.99 (0.70–1.39)	0.9			25.8		1.42 (0.94–2.16)	0.1			45.4		1.41 (0.99–2.01)	0.05		
occupational status																			
active	670	63.4	**<0.001**	1.70 (1.33–2.18)	<0.001	1.84 (1.35–2.52)	**<0.001**	27.9	**<0.001**	1.97 (1.45–2.68)	<0.001	1.60 (1.07–2.40)	**0.02**	49.6	**<0.001**	1.77 (1.38–2.27)	<0.001	1.79 (1.28–2.51)	**<0.001**
passive	420	50.5		ref.		ref.		16.4		ref.		ref.		35.7		ref.		ref.	
self-declared financial status																			
good	510	62.2	0.06	1.41 (0.98–2.02)	0.06			28.0	**0.004**	1.71 (1.09–2.67)	0.02	1.26 (0.77–2.08)	0.4	47.1	0.07	1.54 (1.07–2.23)	0.02	1.02 (0.68–1.55)	0.9
moderate	424	55.7		1.08 (0.74–1.56)	0.7			19.8		1.08 (0.68–1.73)	0.7	0.88 (0.52–1.49)	0.6	43.6		1.34 (0.92–1.96)	0.1	0.91 (0.60–1.39)	0.7
bad	156	53.8		ref.				18.6		ref.		ref.		36.5		ref.		ref.	
Medical																			
wearing spectacles or contact lenses																			
yes	755	58.0	0.7	0.94 (0.73–1.23)	0.7			23.8	0.7	1.07 (0.79–1.45)	0.7			45.6	0.2	1.20 (0.92–1.55)	0.2		
no	335	59.4		ref.				22.7		ref.				41.2		ref.			
eye condition diagnosed by a doctor																			
yes	386	63.7	**0.009**	1.41 (1.09–1.82)	0.009	1.45 (1.11–1.90)	**0.007**	29.8	**<0.001**	1.69 (1.27–2.26)	**<0.001**	1.94 (1.43–2.62)	**<0.001**	51.3	**<0.001**	1.56 (1.21–2.00)	<0.001	1.67 (1.28–2.17)	**<0.001**
no	704	55.5		ref.		ref.		20.0		ref.		ref.		40.3		ref.		ref.	
daily outdoor exposure during the spring and summer months																			
<1 h	220	57.3	0.06	1.22 (0.84–1.77)	0.3	0.96 (0.65–1.43)	0.9	25.9	**0.001**	2.07 (1.29–3.32)	0.003	1.77 (1.09–2.88)	**0.02**	40.5	0.4	ref.			
1–4 h	635	61.1		1.43 (1.06–1.93)	0.02	1.18 (0.85–1.62)	0.3	26.0		2.08 (1.39–3.11)	<0.001	1.81 (1.19–2.75)	**0.05**	44.9		1.20 (0.88–1.64)	0.3		
≥5 h	235	52.3		ref.		ref.		14.5		ref.		ref.		46.0		1.25 (0.86–1.82)	0.2		

Bolded are statistically significant values.

Age 50–59 years was associated with lower odds of use (aOR = 0.65, 95% CI: 0.44–0.97, p = 0.04).

For cloudy conditions significantly associated with higher odds of sunglasses use were:

– younger age (18–29 years: aOR = 2.10, 95% CI: 1.25–3.53, p = 0.005),
– occupational activity (aOR = 1.60, 95% CI: 1.07–2.40, p = 0.02),
– having a diagnosed eye condition (aOR = 1.94, 95% CI: 1.43 – 2.62, p < 0.001),
– shorter daily outdoor exposure (≤4 h, p < 0.05).

### Factors associated with the use of sunglasses with UV protection

Significant sociodemographic differences in the use of sunglasses with UV protection were observed (Table 4). In multivariable logistic regression, older age was significantly associated with higher odds of using sunglasses with UV protection, particularly among individuals aged 40–49 years (aOR = 1.71, 95% CI: 1.08–2.72, p = 0.02), 50–59 years (aOR: 1.93; 95% CI: 1.22–3.05, p = 0.005), and ≥60 years (aOR = 1.61, 95% CI: 1.01–2.57, p = 0.04), compared to those aged 18–29 years. Higher education (aOR = 1.49, 95% CI: 1.15–1.94, p = 0.002), occupational activity (aOR = 1.79, 95% CI: 1.28–2.51, p < 0.001), living in the largest cities (≥500 000 residents: aOR = 1.79, 95% CI: 1.20–2.68, p = 0.005), and having a doctor-diagnosed eye condition (aOR = 1.67, 95% CI: 1.28–2.17, p < 0.001) were also independently associated with higher odds of use (Table 4).

### Sociodemographic differences in knowledge of UVR and eye protection

Significant sociodemographic differences in knowledge of UVR and eye protection were observed (Table 5). Higher levels of knowledge were most consistently observed among respondents with higher education (p < 0.05), those wearing spectacles or contact lenses (p < 0.05), those using sunglasses with UV protection (p < 0.05), and those with eye conditions diagnosed by a doctor (p < 0.05). Moreover, for most of the analyzed questions, older respondents more often provided correct answers (Table 5).

**Table 5.** Socio-demographic differences in knowledge of ultraviolet (UV) radiation (UVR) and eye protection, nationwide cross-sectional survey, Poland, 2026

Variable	Respondents [n]	Agreement level													
		rather agree or strongly agree												rather disagree or strongly disagree	
		UVR can still reach the eyes on a cloudy day		reflection of sunlight from snow increases eye exposure to UVR		UVR exposure may be higher in mountainous areas than at lower altitudes		UV protection in sunglasses depends primarily on the filter (e.g., UV400), not on the level of lens tint		long-term exposure to UVR may contribute to the development of certain eye diseases		intense UV exposure (e.g., in mountains or on snow) may lead to acute damage to the eye surface (“snow blindness”)		darker lenses always provide better protection against UVR	
		%	p	%	p	%	p	%	p	%	p	%	p	%	p
Socioeconomic															
gender															
female	590	76.1	0.09	78.6	**0.009**	75.4	0.8	70.7	0.9	78.0	0.1	75.8	0.6	27.3	0.2
male	500	71.6		71.8		74.8		70.4		73.8		77.2		24.2	
age															
18–29 years	139	75.5	0.2	61.9	**<0.001**	64.0	**0.002**	64.0	0.2	71.2	0.07	67.6	**0.01**	27.3	**<0.001**
30–39 years	220	79.5		76.4		71.8		70.0		78.2		77.3		35.5	
40–49 years	205	75.1		74.1		77.6		68.3		71.2		72.2		30.7	
50–59 years	203	70.0		74.4		74.6		72.4		74.9		77.8		22.7	
≥60 years	323	71.5		82.4		80.8		74.0		80.5		81.4		17.6	
higher education															
yes	500	82.0	**<0.001**	80.4	**<0.001**	80.6	**<0.001**	73.6	**0.04**	79.6	**0.01**	81.8	**<0.001**	35.2	**<0.001**
no	590	67.3		71.4		70.5		68.0		73.1		71.9		18.0	
currently married															
yes	586	74.6	0.7	78.3	**0.02**	78.8	**0.002**	72.2	0.2	77.1	0.4	78.2	0.1	25.8	0.9
no	504	73.4		72.2		70.8		68.7		74.8		74.4		26.0	
place of residence															
rural area	432	71.1	0.1	72.5	0.08	72.5	0.2	68.8	0.4	73.4	0.1	74.3	0.2	24.3	0.1
city															
<20 000 residents	142	78.2		81.7		80.3		75.4		80.3		76.1		22.5	
20 000–99 999 residents	207	72.9		73.9		73.4		68.1		73.4		75.4		24.2	
100 000–499 999 residents	171	73.1		74.9		75.4		70.2		76.6		76.6		28.1	
≥500 000 residents	138	81.9		81.9		80.4		75.4		83.3		84.8		34.1	
having children <18 years															
yes	275	78.5	**0.04**	72.7	0.2	76.4	0.6	67.6	0.2	74.5	0.5	72.7	0.1	31.6	**0.01**
no	815	72.5		76.4		74.7		71.5		76.6		77.7		23.9	
number of household members															
1 (living alone)	178	70.8	0.5	71.9	**0.03**	71.9	0.3	68.5	0.8	73.6	**0.04**	74.2	**0.02**	25.8	0.5
2	397	74.1		80.1		77.6		71.3		80.4		81.1		23.9	
≥3	515	75.1		73.2		74.4		70.7		73.6		73.6		27.4	
occupational status															
active	670	77.2	**0.003**	75.8	0.8	75.5	0.7	71.9	0.2	76.4	0.7	77.6	0.2	28.5	**0.01**
passive	420	69.0		75.0		74.5		68.3		75.5		74.5		21.7	
self-declared financial status															
good	510	77.8	**0.03**	75.9	0.2	75.1	0.05	69.4	0.6	75.5	0.5	76.5	0.3	26.7	0.6
moderate	424	70.5		77.1		77.8		72.2		77.8		78.1		24.3	
bad	156	71.2		69.9		67.9		69.9		73.1		71.8		27.6	
Medical															
wearing spectacles or contact lenses															
yes	755	76.2	**0.02**	78.1	**0.002**	78.3	**<0.001**	73.0	**0.008**	78.0	**0.02**	79.2	**0.001**	23.8	**0.02**
no	335	69.3		69.6		68.1		65.1		71.6		70.1		30.4	
eye condition diagnosed by a doctor															
yes	386	78.0	**0.03**	80.8	**0.002**	78.8	**0.04**	75.6	**0.006**	79.0	0.09	82.4	**<0.001**	24.6	0.5
no	704	71.9		72.6		73.2		67.8		74.4		73.2		26.6	
daily outdoor exposure during the spring and summer months															
<1 h	220	75.0	**0.04**	72.7	0.2	74.1	0.3	69.5	0.9	77.3	0.4	76.8	0.6	27.7	0.1
1–4 h	635	76.1		77.6		76.7		71.2		76.9		77.2		27.1	
≥5 h	235	67.7		72.3		71.9		69.8		72.8		74.0		20.9	
using sunglasses with UV protection															
yes	482	79.0	**<0.001**	80.1	**<0.001**	79.9	**<0.001**	76.6	**<0.001**	80.1	**<0.001**	80.4	**<0.001**	27.3	0.2
no	608	66.3		68.4		67.7		61.1		69.8		70.3		23.7	

Bolded are statistically significant values.

## DISCUSSION

This study provides a comprehensive assessment of public behaviors towards the sunglasses use, UV protection as well as knowledge on UV-related environmental risks for eye health among adults in Poland [3–6]. Over half of respondents reported frequent use of sunglasses on sunny days in spring and summer, with only one-quarter of respondents using sunglasses on cloudy days, which suggest that preventive behaviors are dependent on perceived sunlight rather than actual UV exposure. However, only 44.2% of respondents declared use of sunglasses with adequate UV filters that provides protection against UV-A and UV-B radiation [3,4]. Approximately three-quarters of respondents presented a good level of awareness of selected aspects of UVR, but misperception of lenses darkness and its impact on UV protection still occurs. This study also revealed sociodemographic differences in preventive behaviors related to UVR by age, educational level, occupational status, place of residence and history of eye conditions diagnosed by a doctor.

In this study, use of sunglasses during different weather conditions (sunny and cloudy days) as well as different seasons (spring and summer/winter and autumn) were analyzed. Findings from this study revealed that in spring and summer, the use of sunglasses is >2 times more prevalent on sunny days (58.5% respondents), than on cloudy days (23.5% respondents). These findings are consistent with previously published data from Germany, Poland, and Jordan, which have shown that sunglasses use is often inconsistent and strongly influenced by weather conditions and subjective perception of risk rather than objective exposure levels. Individuals are less likely to use eye protection on cloudy days, despite continued UV exposure [15–17]. Similar behavior was reported in previous surveys, including a Polish study among university students in which sunglasses were one of the most commonly used photoprotective measures, but consistent year-round protection was uncommon, and a German nationwide survey showing that only 50.6% of adults wore sunglasses always or often in summer, with even lower use among outdoor workers and children [15,16].

This study also provides important self-reported data on the use of sunglasses with UV filters that is the most effective way to protect eyes against UVR [3,9–13]. In this study, only 44.2% of respondents reported using sunglasses with UV protection, while 22.3% were unsure whether their sunglasses provided such protection. This observation points out significant gaps in public awareness of effective UV protection methods and the role of adequate UV filters in sunglasses. The authors can hypothesize that some individuals who use sunglasses may be not aware that their sunglasses do not provide sufficient UV protection and UV-related health risk for eye health are still present [17]. Findings from this study are in line with previous international reports showing that consumer awareness of the UV-filtering capacity of sunglasses remains incomplete, while the actual protective performance of sunglasses depends on lens quality, frame design, and fitting, and may be insufficient in some commercially available products [17,18].

Among those who reported not using sunglasses with UV protection, the main reason for this behavior was lack of perceived need reported by 81.4% of respondents. This highlights the need for educational campaigns on eye health, especially health risk caused by environmental factors [27,28]. Economic difficulties related to acquiring sunglasses with UV filters were reported by 16.4% of respondents that suggest a limited impact of economic factors in preventive behaviors against UVR. Uncertainty about which products are reliable was reported by approx. one-tenth of respondents, highlighting the need for educational campaigns that not only raise awareness but also provide practical guidance on how to select sunglasses with adequate UV protection [17,27,28].

In this study, sociodemographic differences in both behaviors and knowledge were identified. Higher use of sunglasses and UV protection was associated with female gender, occupational activity, urban residence, and higher education, which is in line with previous research indicating that health-promoting behaviors are more common among individuals with higher socioeconomic status [29]. Moreover, better knowledge on UVR was observed among those with higher education, individuals using spectacles or contact lenses as well as diagnosed with eye conditions. This observations suggest that prior contact with healthcare system (especially ophthalmologist) plays an important role in shaping public attitudes towards preventive behaviors against UVR [13,25].

### Practical implications

This study has several practical implications for ophthalmology and public health. The relatively low prevalence of the use of sunglasses with certified UV protection and the persistence of misconceptions regarding UV protection highlight the need for educational initiatives promoting evidence-based eye protection. These campaigns should emphasize that harmful ocular UV exposure occurs not only on sunny days but also during cloudy weather and should provide practical guidance on selecting sunglasses with certified UV protection (e.g., UV400).

Ophthalmologists, optometrists, and occupational health professionals should play a more active role in educating patients about UV-related eye hazards and appropriate preventive measures. Moreover, the observed sociodemographic differences suggest that public health interventions should be tailored to groups with lower awareness and less frequent use of sunglasses, particularly younger adults, individuals without higher education, and those who are occupationally inactive.

Public health campaigns should also promote the use of the WHO Global Solar UV Index [30] as a simple and evidence-based tool to increase awareness of daily UV exposure and encourage appropriate eye protection behaviors, including the use of sunglasses with certified UV protection.

### Limitations

This study has several limitations. First, the cross-sectional design based on self-reported data may be subject to recall bias. The scope of questions was limited to the most important ones identified in previously published studies as well as refined during expert consultation. Data were collected using the CAWI technique, which may have excluded individuals without internet access or with lower digital literacy. Actual UV exposure levels were not objectively measured. Moreover, information regarding the use of sunglasses with UV protection was based entirely on participants' self-reports and was not objectively verified by the research team. Therefore, some respondents may have incorrectly assumed that their sunglasses provided adequate UV protection or may have been unaware of the actual protective properties of the lenses.

## CONCLUSIONS

The use of sunglasses under cloudy conditions was infrequent despite generally good awareness of UVR, suggesting a gap between knowledge and preventive behaviors. Less than half of respondents reported using sunglasses with UV protection, and misconceptions about effective protection remain common. Sociodemographic differences were observed, with higher use of sunglasses and better knowledge of UV protection more common among older individuals, those with higher education, residents of urban areas, occupationally active respondents, and individuals with diagnosed eye conditions. These patterns suggest that both access to information and personal health experience may influence protective behaviors. The findings may inform the development of targeted public health interventions to promote evidence-based eye protection behaviors, particularly among groups with lower levels of knowledge and less frequent use of sunglasses.
